# Research on Public Fitness Equipment Experience Based on Satisfaction

**DOI:** 10.3390/healthcare9050501

**Published:** 2021-04-26

**Authors:** Zufang Zheng, Jiancheng Mo, Yuanyuan Xu

**Affiliations:** School of Industrial Design, Hubei University of Technology, Wuhan 430068, China; 20140094@hbut.edu.cn (Z.Z.); yy2396033986@gmail.com (Y.X.)

**Keywords:** public fitness equipment, experience design, satisfaction, IPA model, FAHP

## Abstract

In order to improve the user experience of public fitness equipment, based on the principles of experience design principle, the Norman 3-level theory is used to obtain product materials, using safety, equipment practicality, product interest, and humanistic construction experience. The 5-level Likert evaluation scale is used to conduct a satisfaction survey on the index factors. According to the score, the fuzzy comprehensive evaluation method is applied to calculate the weights and average satisfaction values of the first and second indexes and discover the practical characteristics, interesting characteristics, and humanities of users. The characteristic satisfaction is low. The IPA model is used to analyze and evaluate the position of each evaluation index factor quadrant, and the experience factors that need improvement are obtained. In view of the analysis results, suggestions for advancement of public fitness equipment and factors in urgent need of improvement are put forward, and the safety experience refine design of the stroller is provided, which offers an effective reference for the experience design of public fitness equipment.

## 1. Introduction

Knowing about the satisfaction of users in the process of using fitness equipment and improving the user experience of equipment are crucial for realizing the health strategy of China. Presently, the research on public fitness equipment is biased on a certain factor, such as safety [1]. Yi Yanli uses the “human-thing-human” communication method as the starting point, combining semantics, function, and emotional interaction [2], etc. To explore the new interactive relationship of equipment, Mao Chen uses ergonomic data [3] to explore the functional rationality of equipment and facilities and the optimization of spatial layout, etc. To respond to the refined market demand, the structure of multifunctional and intelligent fitness equipment [4], composite materials [5], man–machine interface [6], emotional needs [7], and usability [8] are also plentiful in research papers. These documents can effectively guide the design and evaluation of a certain field of fitness equipment; however, they are unable to comprehensively judge and optimize from the perspective experience design. Therefore, this paper studies the influencing factors of equipment experience design on user satisfaction and uses the vague analytic hierarchy process (FAHP) [9,10] to establish a comprehensive evaluation model of public fitness equipment user satisfaction to understand the satisfaction of the user’s actual experience process. According to the IPA [11] (importance–performance analysis) quadrant diagram, the importance of the user experience element of the equipment-satisfaction feedback expression proposes the direction and design of optimization and improvement.

## 2. Preparation of Experience Satisfaction Research Materials

### 2.1. Experience Analysis of Public Fitness Equipment

**Figure 1:**
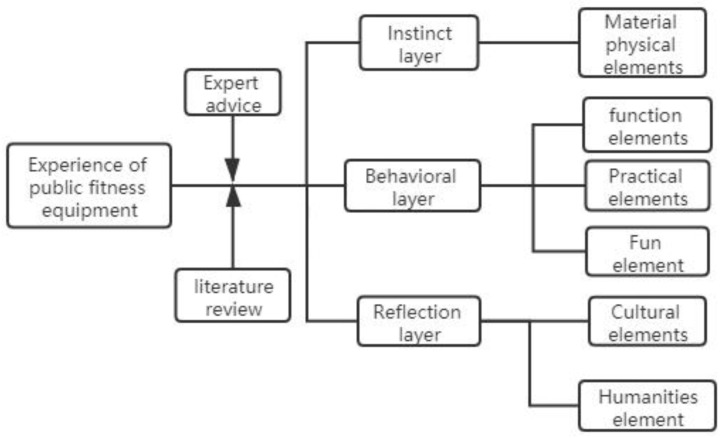
Analysis of the elements of public fitness equipment experience.

Subjective feeling established by the user in the experience of using the product is the user experience of the product, that is, all the feelings before, during, and after use. Norman proposed the three-level principle of experience in “Emotional Design”, namely the instinct level, behavior level, and reflection level. The instinct layer refers to the user’s subjective impressions and feelings about the appearance elements of actual things; the behavior layer refers to the product use process; the user gets the satisfaction and pleasure brought by the product performance, usability, functional practicability, etc.; and the reflection layer focuses on the consciousness and emotional feelings during the process of use, focusing on the experience reflection of humanistic thought and meaning brought by the experience process [12]. Based on the above literature, as shown in Figure 1, preliminary evaluation factors are selected from the physical factors of the product, the user’s behavioral factors, the environmental factors used, and the cultural factors. According to the instinct layer of public fitness equipment experience design, comparative analysis can include the product material characteristics, color, and other intuitive physical factors; the behavior layer includes the safety performance, practicality, interest, and other factors of the product during use; and the reflection layer represents the user’s more advanced ideological experience transforms the entire process of using the product into a special experience, summarizing the cultural nature of the product and the surrounding environment, and the humanistic factors attached to fitness activities.

### 2.2. Public Fitness Equipment User Satisfaction Experiment

Scientific research and evaluation needs to be done to identify whether users are satisfied with the existing public fitness equipment experience or not. Therefore, we have carried out related research work, surveyed and collected user experience satisfaction answers, expounded user experience evaluation factors, research methods, and research results, and analyzed the overall satisfaction of users with public fitness equipment and the satisfaction of various evaluation indicators, as well as their importance. We put forward suggestions for optimization and improvement of public fitness equipment based on the analysis results.

Experimental design and steps:

(1) Investigation of experimental environment

Field investigations were conducted on Changqing Park, Community Poly Park, college fitness venues, and sports parks in Wuhan. It was found that the fitness equipment had a single type, simple functional structure, common equipment aging and damage, and poor comfort experience.

(2) Subject and location

According to the survey, the users of public fitness equipment are mostly parents and children, relatives, and friends of the same age. The age of the test subjects is defined as 6–60 years old, and the satisfaction of adults and children participating in fitness exercises (48–60 years old) is comprehensively considered. The experimental site was a community and sports park in Wuhan. About 30 field survey questionnaires were collected, a 270 receipt data of a network survey questionnaire.

(3) Questionnaire indicators

We referred to the experience and sports literature, focusing on the preliminary selection of evaluation factors from the three aspects of instinct factors, behavioral factors, and reflection factors, and then discussed and analyzed with four industrial design experts, two sports experts, and one material engineering expert. Finally, we determined 23 evaluation indicators.

(4) Data collection and analysis

The questionnaire data was extracted and analyzed for the evaluation index satisfaction, importance, reliability and validity, factor loading analysis, weight analysis of the public fitness equipment experience satisfaction evaluation index, fuzzy comprehensive evaluation, and the final user satisfaction evaluation average, and analysis graph of IPA index of satisfaction.

## 3. Experience Satisfaction Evaluation Index System

### Satisfaction Factor Analysis

A total of 300 questionnaires were distributed in this questionnaire survey, and 287 valid questionnaires were returned, with an effective response rate of 95.6%. The 5-level Likert scale method was used to investigate user satisfaction-importance data on the experience of using public fitness equipment, using SPSS22.0 [13,14] software to perform reliability analysis on the questionnaire and KMO and Bartlett spherical analysis to obtain clones. The Bach reliability coefficient is 0.833. The KMO sample measure value is 0.786, Bartlett’s sphericity test value (based on the exact requirement of the decimal point) is 0.000, indicating that the overall reliability of the survey questionnaire is good, and the original variables are suitable for factor analysis.

First, KMO and Bartlett tests were performed on the sample satisfaction data. When the KMO value is between 0–1, the item value approaches 1, and the Bartlett sphere detection significance *p* value is less than 0.05, it indicates that it is suitable for factor analysis. The test results are shown in Table 1: the KMO value is 0.871, and the Bartlett sphere test corresponds to a *p* value of 0.000, which passes the test.

**Figure 2:**
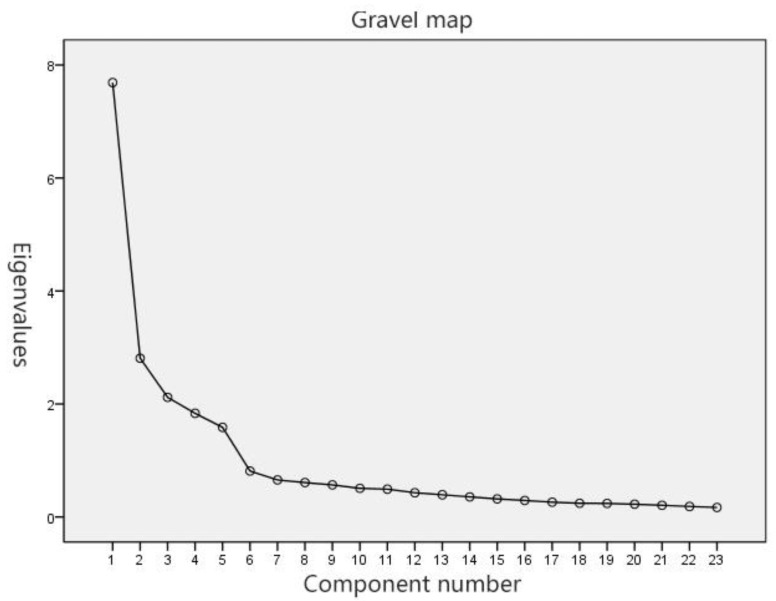
Principal factor extraction graph.

SPSS22.0 software was used to extract the principal component factors and perform exploratory factor analysis on 23 indicators based on the principal factor extraction diagram in Figure 2. It is found that the inflection point is the most obvious at the fifth factor. The first five factors are extracted alond with their contribution to the total variance. The rate reaches 69.72%, which can be better used as a common factor to represent the 23 indicators selected; that is, the 23 indicators of the questionnaire are summarized and classified into five main levels.

After extracting the principal factors, the loading matrix is established. It is found that the common factors of the initial matrix have relatively close loadings on each data index, and it is not easy to analyze the common factor classification. Therefore, the maximum variance method is used to establish the factor loading matrix after orthogonal rotation of the evaluation factor. The processing is shown in Table 2:

Based on the above analysis of satisfaction factors, the questionnaire items are extracted from five common factors, namely the material characteristics of the experience, the safety function characteristics of the equipment, the practical characteristics of the product, the interesting characteristics, and the humanistic characteristics, including 4, 5, 5, 5, and 4, respectively, as well as secondary evaluation factors.

## 4. User Satisfaction Research Methods and Results 

### 4.1. Analysis on the Weight of Satisfaction Evaluation Index

#### Analytic Hierarchy Process

(1) Construct a judgment matrix

The judgment matrix compares the relative importance of the elements of this layer to a certain element of the upper set and uses the 1–9 scale method to obtain the weights of the relative importance of different judgment factors.

(1) $$V=\left[\begin{array}{l} b_{11}\;\;\ldots \;\;b_{1n}\\ \;\;\vdots \;\;\;\;\;\;\;\;\;\;\;\;\;\;\vdots \\ b_{n1}\;\;\ldots \;\;b_{\mathrm{nn}} \end{array}\right]$$

(2) In the formula, bnn—the ratio of element importance in the index layer B and the scheme layer C.

The maximum eigenvalues and eigenvectors of each judgment matrix were calculated, the eigenvectors to obtain the weight ranking were normalized, and the total weight ranking was calculated according to the weight ranking of each level. The steps are as follows:

a. After normalizing the value of the judgment matrix *V*, the eigenvector *W* is obtained by adding and normalizing the rows.
  (2) $$W={\left[W_{1},W_{2},\ldots ,W_{n}\right]}^{T}$$
b. The maximum eigenvalue of the judgment matrix $\lambda_{\max}$ is calculated:
  (3) $$\lambda_{\max}=\frac{\sum_{i=1}^{n}OW_{i}}{nW_{i}}$$
  where, $OW_{i}$—Matrices *O* and *W* take the opportunity.

(3) A consistency check was performed on the weight vector. Due to the incomplete understanding of things, the error of judgment increases with the increase of the value of n. It is necessary to implement the consistency of the judgment matrix and the consistency of the random judgment detection logic. The standardization steps are as follows:
  a. Calculate *CI* (Consistency Index)
    (4) $$CI=\left(\lambda_{\max}-n\right)/\left(n-1\right)$$
  b. Calculate *RI* (Average Random Consistency Index): *RI* is the arithmetic average value obtained after repeated calculation of the eigenvalues of the random judgment matrix. Table 3 lists the *RI* values of 1–10:
  c. Calculate *CR*:
    (5) CR=CI/RI

When *CR* < 0.1, it indicates that the consistency of the judgment matrix is better.

(4) The calculation results of the weight of satisfaction at all levels were solved by programming in Python [15], and the weight values of all levels of indicators were obtained, as shown in Table 4:

The consistency test was carried out on the index layer and the scheme layer, respectively, and the consistency ratio *CR* was less than 0.1. The results are shown in Table 5:

### 4.2. Fuzzy Comprehensive Evaluation

According to the fuzzy evaluation model, the membership matrix R of five evaluation indicators of material properties, safety function properties, practical properties, interesting properties, and humanistic properties is constructed as follows:

(6) $$R=\left[\begin{array}{l} r_{11}\;\;\;\;r_{12}\;\ldots \;r_{1m}\\ r_{21}\;\;\;\;r_{22}\;\ldots \;r_{2m}\\ \;\;\vdots \;\;\;\;\;\;\;\;\vdots \;\;\;\ldots \;\;\;\vdots \\ r_{n1}\;\;\;\;r_{n2}\;\ldots \;r_{\mathrm{nm}} \end{array}\right]$$

r_ij_ represents the degree of membership of the influencing factor *U_i_* to the level *V_i_* [16], r_ij_ ∈ [0,1].

$R_{1}=\left[\begin{array}{l} 0.6\;\;0.3\;\;0.1\;\;0\;\;0\\ 0.4\;\;0.4\;\;0.2\;\;0\;\;0\\ 0.3\;\;0.7\;\;\;\;0\;\;\;\;0\;\;0\\ 0.5\;\;0.3\;\;0.2\;\;0\;\;0 \end{array}\right]$ $R_{2}=\left[\begin{array}{l} 0.4\;\;0.3\;\;0.2\;\;0.1\;\;0\\ 0.3\;\;0.6\;\;0.1\;\;\;\;0\;\;\;\;0\\ 0.5\;\;0.3\;\;0.2\;\;\;\;0\;\;\;\;0\\ 0.6\;\;0.2\;\;0.2\;\;\;\;0\;\;\;\;0\\ 0.4\;\;0.3\;\;0.2\;\;0.1\;\;0 \end{array}\right]$

$R_{3}=\left[\begin{array}{l} 0.3\;\;0.6\;\;0.1\;\;\;\;0\;\;\;\;0\\ \;\;0\;\;\;\;0.7\;\;0.2\;\;0.1\;\;0\\ 0.1\;\;0.6\;\;0.3\;\;\;\;0\;\;\;\;0\\ 0.2\;\;0.5\;\;0.2\;\;0.1\;\;0\\ 0.4\;\;0.3\;\;0.3\;\;\;\;0\;\;\;\;0 \end{array}\right]$ $R_{4}=\left[\begin{array}{l} 0.3\;\;0.5\;\;0.1\;\;0.1\;\;0\\ 0.2\;\;0.4\;\;0.2\;\;0.2\;\;0\\ 0.3\;\;0.4\;\;0.3\;\;\;\;0\;\;\;\;0\\ 0.1\;\;0.6\;\;0.3\;\;\;\;0\;\;\;\;0\\ \;\;0\;\;\;\;0.4\;\;0.4\;\;0.2\;\;0 \end{array}\right]$

$R_{5}=\left[\begin{array}{l} 0.2\;\;0.5\;\;0.3\;\;\;\;0\;\;\;\;0\\ 0.2\;\;0.5\;\;0.3\;\;\;\;0\;\;\;\;0\\ 0.4\;\;0.3\;\;0.1\;\;0.2\;\;0\\ 0.5\;\;0.3\;\;0.2\;\;\;\;0\;\;\;\;0 \end{array}\right]$

Fuzzy comprehensive evaluation calculation for each index layer:

*B*_1_ = *W*_1_ × *R*_1_ = (0.4752, 0.3915, 0.1333, 0, 0)

*B*_2_*= W*_2_ × *R*_2_ = (0.4552, 0.3158, 0.1884, 0.0406, 0)

*B*_3_*= W*_3_ × *R*_3_ = (0.1917, 0.5267, 0.2121, 0.0494, 0)

*B*_4_*= W*_4_ × *R*_4_ = (0.2221, 0.4482, 0.2111, 0.1186, 0)

*B_5_ = W_5_* × *R_5_* = (0.3393, 0.371, 0.1813, 0.1084, 0)

De-fuzzy calculation of each evaluation set to obtain the evaluation value:

*P*_1_ = 5b_11_ + 4b_12_ + 3b_13_ + 2b_14_ + 1b_15_ = 4.3419

*P*_2_ = 5b_21_ + 4b_22_ + 3b_23_ + 2b_24_ + 1b_25_ = 4.1856

*P*_3_ = 5b_31_ + 4b_32_ + 3b_33_ + 2b_34_ + 1b_35_ = 3.8004

*P*_4_ = 5b_41_ + 4b_42_ + 3b_43_ + 2b_44_ + 1b_45_ = 3.7738

*P_5_* = 5b_51_ + 4b_52_ + 3b_53_ + 2b_54_ + 1b_55_ = 3.9412

In the same way, the fuzzy evaluation index of the target layer is calculated:

*A* = *W* × *B* = (0.3842, 0.3977, 0.1672, 0.0489, 0)

*P_final_* = 5 × 0.3842 + 4 × 0.3977 + 3 × 0.1672 + 2 × 0.0489 + 1 × 0 = 4.1113

It can be seen from the evaluation result that the final score of the evaluation is 4.1113 points. The evaluation level is low, indicating that users’ overall satisfaction with public fitness is low, and further in-depth research and analysis from the perspective of experience satisfaction (IPA) are needed. The above results can be summarized as material property B1, and the comprehensive evaluation of safety function property B2 is slightly higher than the overall average score, indicating that these two aspects are slightly satisfactory. At the same time, the scores of practical feature B3, fun feature B4, and humanistic feature B5 are lower than the overall score, indicating that there is room for improvement in these three aspects.

### 4.3. IPA Analysis of Satisfaction of Public Fitness Equipment Experience

Through the normal distribution test on satisfaction and importance, it is found that satisfaction and importance do not conform to the normal distribution; therefore, the paired sample rank sum test is used. The body feel of the material, the cleanliness of the material, the safety and anti-pinch property, the safety limit, the safety buffer measures, the professionalism of the equipment, the shading, the interaction of the equipment, the appearance of the product, and the incentive system of fitness activities can be seen in Table 2. The significance *p* value of is less than 0.05, indicating that there is a significant difference in satisfaction and importance.

**Figure 3:**
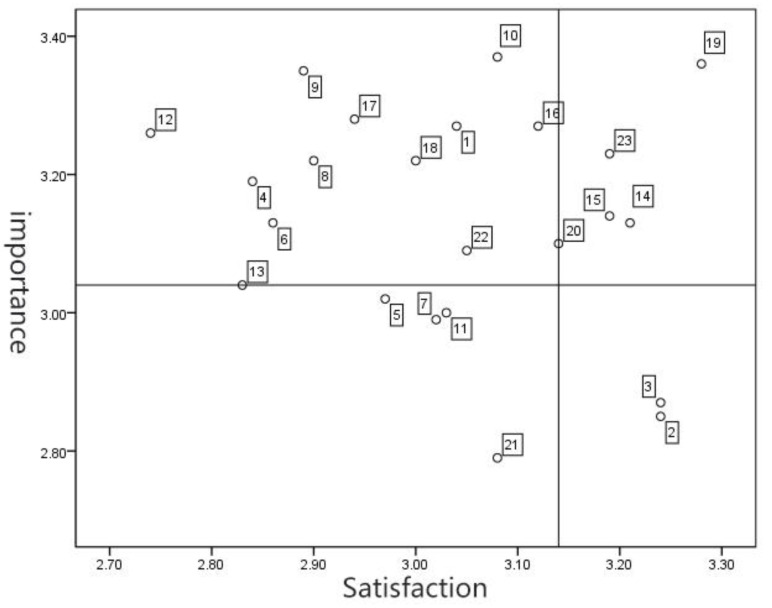
IPA analysis of public fitness equipment experience satisfaction index.

IPA model analysis, namely the importance-satisfaction four-quadrant performance method, to establish a coordinate system with satisfaction and importance as the x and y axes and build the IPA analysis chart, as shown in Figure 3, with the mean satisfaction factor 3.14 and the mean importance factor 3.04, divides the coordinate system into a continuous maintenance area, a follow-up opportunity area, an advantage improvement area, and an urgent improvement area, which can clearly detect the user’s satisfaction with the public fitness equipment experience and the quadrant of each evaluation factor and obtain the improvement design factor.

Figure 3 shows the satisfaction and importance values represented by each factor in this satisfaction evaluation. The results of the four-quadrant analysis are as follows:

(1) The area in urgent need of improvement (high importance, low satisfaction) includes the evaluation factors: material feel, material cleanliness, safety and anti-pinch, safety limit, safety buffer measures, equipment professionalism, equipment sunshade, rainproof, human–computer interaction experience (the definition of human-computer interaction in the field of industrial design has evolved from the interaction between humans and electronic products, including the overall interactive experience of users and equipment products), equipment interaction, product appearance, and cultural exchange.
(2) The advantage improvement area (high importance, high satisfaction) includes judging factors: equipment diversity operation innovation, site selection, expression of regional culture, color beauty, and ease-of-learning equipment.
(3) The follow-up opportunity zone (low importance, low satisfaction) includes the evaluation factors: safety anti-collision, safety anti-skid, equipment man-machine design, and the motivation system of fitness activities.
(4) The maintenance zone (low importance, high satisfaction) includes the evaluation factors: weather resistance of materials and durability of materials.

## 5. Optimized Design of Public Fitness Equipment Experience

### 5.1. Optimization Suggestion and Design Direction

After analyzing the overall satisfaction of public fitness equipment and then analyzing the refined index of experience satisfaction IPA, certain suggestions and improvement directions are put forward for the factors of public fitness equipment experience satisfaction being in the improvement zone.

(1) In terms of material experience, it is advisable to consider the use of high-tech composite materials, modified plastics, composite use of multiple materials, etc., using dust-proof and waterproof materials or dust-proof and hydrophobic coatings for contact-use parts, which not only ensures the safety and stability of the equipment but also has better elasticity and flexibility at the same time, and the experience is gentler and more comfortable.
(2) In terms of practicality and safety experience, using ergonomics theory, combing the user’s human–machine size, sensory experience, and psychological corrections, considering the coordination and unity of man–machine environment, as well as the safety protection of equipment in the initial design of the product, such as the design of parts package, setting the angle of the rotating shaft, sliding buffer, and shock absorption measures, so as to reduce the possible injury to the user caused by the equipment itself and the place.
(3) In terms of fun experience, seeking innovative ways of operation, the diversity of equipment types, not limited to traditional colors and shapes, explores more vivid and interesting product appearances, considers new intelligent human–computer interaction experience, and pays attention to the linkage interaction between equipment. While ensuring a private space, fitness activities make a fun entertainment experience.
(4) In terms of humanistic experience, there should be a combing national culture appropriately according to regional characteristics, creating characteristic equipment user experience or scene cultural experience, developing national leisure sports projects, enhancing users’ awareness and innovative experience of traditional culture, organizing fitness activities, and regularizing fitness activities to enhance the user’s participation experience.

### 5.2. Improved Design of Stroller

The survey results show that 79.51% of people are most willing to use the walker for leisure exercise, and the safety problems of the walker are also more obvious. According to the previous research conclusions, this article mainly proposes an improvement plan for the safety performance of the walker.

**Figure 4:**
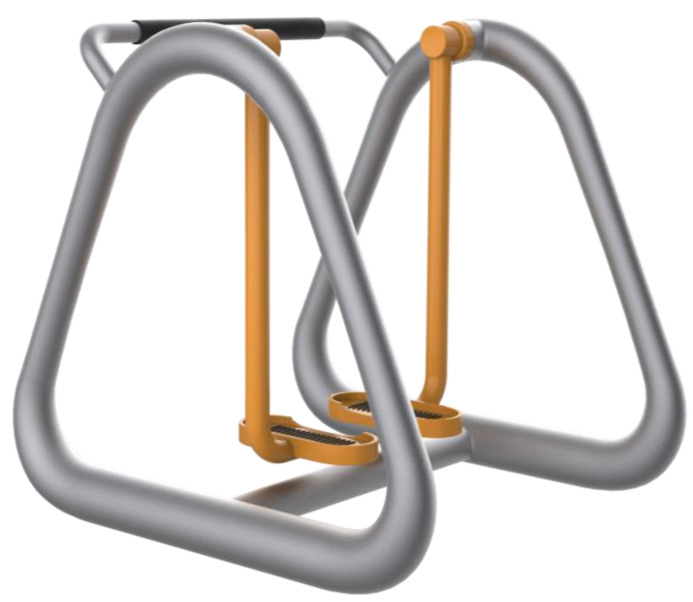
The overall structure of the walker.

Among the factors affecting the safety performance of the walker, the pendulum movement that is most likely to cause safety accidents is selected to optimize the failure events of itself and others due to the excessive amplitude [17]. As shown in Figure 4, the whole walker adopts a cone-shaped design to increase stability while clarifying the boundaries of the activity interval, distinguishing the pedestrian and user behavior areas, and reducing the safety hazards of accidentally hurting pedestrians.

**Figure 5:**
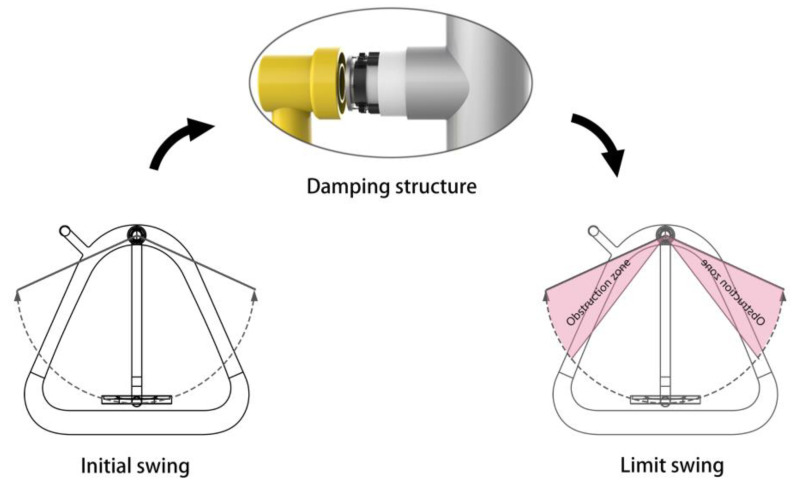
Improved design of stroller safety experience.

**Figure 6:**
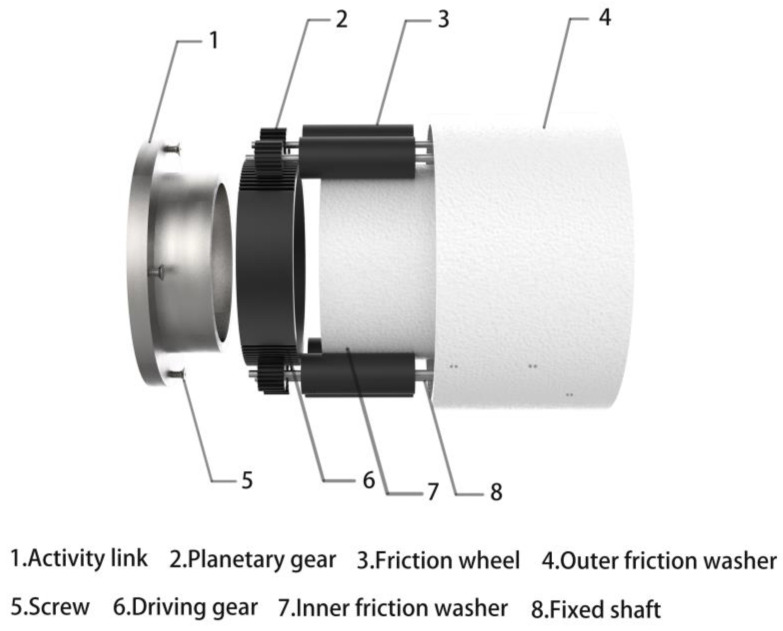
Disassembly of the damping structure.

The patent CN201520877546.5 [18] is adapted to the design. Are shown in Figure 5 and Figure 6 shows, the rotating shaft of the stroller is equipped with a mechanical damping structure. The swing movable part is equipped with a driving gear with cog on the upper and lower sides. The rear stationary part is equipped with planetary gears on the left and right sides. The planetary gears are connected when the friction wheel is in contact with the friction part of the inner wall cavity of the stationary part. When the swing part swings up to a certain angle, the driving gear and the planetary gear mesh to drive the friction wheel to rotate along the friction inner wall, generating frictional resistance to inhibit the swinging part of the equipment from continuing to rise. The greater the rising angle of the swing part of the damping device, the more gears are engaged; that is, the more the friction wheel runs, the greater the resistance. The device is a speed-up gear ratio. The faster the driving gear speed, the more the friction wheel turns, and the greater the resistance generated. It can better solve the safety hazard caused by the excessive rotation of the rotating part.

## 6. Conclusions

In order to improve the user experience of public fitness equipment, guided by the concept of user satisfaction, the evaluation factors of public fitness equipment experience are determined through literature research and expert opinions, and factor analysis and fuzzy evaluation methods are used to determine the user’s satisfaction with public fitness equipment experience. The IPA analysis method classifies and analyzes each evaluation factor. The results show that users’ overall satisfaction with public fitness equipment is low; they are dissatisfied with the evaluation indicators of practical, interesting, and humanistic characteristics; and they have low satisfaction with most of the evaluation factors. According to the IPA analysis chart, this paper puts forward the optimization suggestions and directions of each factor in the five evaluation indexes and makes the experience optimized design for the safety problems existing in the walkers, which provides a certain reference for related public fitness equipment experience design research.

## Figures and Tables

**Table 1:** KMO and Bartlett sphere inspection.

Kaiser–Meyer–Olkin Measure of Sampling Adequacy.		0.871
Bartlett’s Test of Sphericity	Approx. Chi-Square	3147.943
	df	253
	Sig.	0.000

**Table 2:** Extraction results of satisfaction evaluation factors.

Evaluation Factor	Factor Loading	Paired Rank Sum and Variance	
Common Factor 1: Material Characteristics		*p*-Value	Variance Contribution Rate %
Material feel	0.853	0.047	15.244
Weather resistance of materials	0.851	0.193	
Durability of materials	0.765	0.890	
Material cleanliness	0.833	0.002	
Common factor 2: Safety features			14.068
Safety and anti-collision	0.762	0.425	
Anti-pinch	0.728	0.034	
Slip resistance	0.779	0.619	
Safety limit	0.830	0.010	
Safety buffer measures	0.816	0.000	
Common factor 3: Product practical characteristics			13.848
Equipment professionalism	0.718	0.009	
Equipment man-machine design	0.742	0.949	
Shading	0.747	0.000	
Rainproof	0.777	0.063	
Easy to learn equipment	0.695	0.431	
Common factor 4: Interesting characteristics			13.421
Color aesthetics	0.780	0.712	
Human-computer interaction experience	0.772	0.243	
Interactivity	0.756	0.015	
Product appearance	0.771	0.044	
Operational innovation	0.649	0.558	
Common factor 5: Humanistic characteristics			13.139
Embodiment of regional culture	0.852	0.785	
Incentive system	0.801	0.012	
Cultural communication	0.862	0.678	
Site selection	0.859	0.734	

**Table 3:** Values of *RI*.

Dimension	*RI*	Dimension	*RI*
1	0	11	1.52
2	0	12	1.54
3	0.52	13	1.56
4	0.89	14	1.58
5	1.12	15	1.59
6	1.26	16	1.5943
7	1.36	17	1.6064
8	1.41	18	1.6133
9	1.46	19	1.6207
10	1.49	20	1.6292

**Table 4:** Weights of public fitness equipment experience evaluation index system.

Target Layer	Index Layer	Weights	Scheme Layer	Weights
Experience of public fitness equipment	Material characteristicsB_1_	0.439	Material experienceC_14_	0.383
			Weather resistanceC_15_	0.347
			DurabilityC_16_	0.142
			CleanlinessC_17_	0.128
	Safety featuresB_2_	0.106	CrashworthinessC_18_	0.171
			Anti-pinchC_19_	0.116
			Slip resistanceC_20_	0.288
			Safety limitSafety limitC_21_	0.19
			Buffer measuresC_22_	0.235
	Product practical B_3_	0.099	Equipment professionalismC_23_	0.092
			Man-machine designC_24_	0.293
			ShadingC_25_	0.139
			RainproofC_26_	0.201
			Easy to learnC_27_	0.275
	Interesting characteristicsB_4_	0.106	Color aesthetics C_28_	0.308
			Man–machine interactionC_29_	0.356
			InteractivityC_30_	0.166
			AppearanceC_31_	0.087
			Operational innovationC_32_	0.083
	Humanistic characteristicsB_5_	0.250	Embodiment of regional culture C_33_	0.221
			Incentive systemC_34_	0.134
			Cultural communication C_35_	0.542
			Site selection C_36_	0.103

**Table 5:** Consistency test.

Level	*λ_max_*	*CI*	*CR*
Index layer	5.219	0.055	0.049
Scheme layer 1	4.021	0.007	0.008
Scheme layer 2	5.338	0.084	0.075
Scheme layer 3	5.141	0.035	0.031
Scheme layer 4	5.327	0.082	0.073
Scheme layer 5	4.238	0.079	0.089

## Data Availability

Data is reflected in the article and is only applicable to the research in this article. Data sharing is not applicable to this article.

## References

[1] Yu H., Hu J., Liu G. (2020). Safety analysis and research on public outdoor fitness equipment. Contemp. Sports Sci. Technol..

[2] Yi Y. (2013). Research on the Interactive Design of Outdoor Fitness and Entertainment Facilities. Master’s Thesis.

[3] Mao C. (2016). Research on the Design of Northern Outdoor Fitness Equipment Based on Man-Machine Environment. Master’s Thesis.

[4] Yu W., Wang L., Wang X., Niu Y., Chen M., Xue W., Wei J. (2020). Structural design and finite element analysis of home-type multifunctional treadmills. Mech. Des..

[5] Zheng Y. (2020). The application of plastic composite materials in sports facilities and fitness equipment—comment on “Sporting Goods and Equipment Standards Compilation”. Mater. Prot..

[6] Zhao C., Li J., Ren J., Xue A. (2019). Research on Human-Machine Interface Evaluation Method of Fitness Equipment Based on Multi-factor Fusion. J. Graph..

[7] Yang A., Wang Q., Zhu N., Fu N. (2017). Research on the shape design of elderly exercise bikes based on emotional needs. Mech. Des..

[8] Lu C., Xiao Z., Fu X. (2020). User experience design of household integrated stove based on Kano model. Packag. Eng..

[9] Averill A.F. (2020). The usefulness and application of fuzzy logic and fuzzy AHP in the materials finishing industry. Trans. IMF.

[10] Yan L., Claudia M.E. (2020). A review of fuzzy AHP methods fordecision-making with subjective judgements. Expert Syst. Appl..

[11] John A.M., John C.J. (1977). Importance-performance analysis. J. Mark..

[12] Alonso-García M., Pardo-Vicente M.-Á., Rodríguez-Parada L., Nieto D.M. (2020). Do Products Respond to User Desires? Errors and Successes in the Design Process, under the Umbrella of Emotional Design. Symmetry.

[13] Zhou J. (2017). Questionnaire Data Analysis: Six Analysis Ideas for Cracking Apss.

[14] Hong M.C., Xiao C.X. (2012). The Application of SPSS Factor Analysis in the Evaluation of Corporate Social Responsibility. J. Softw..

[15] McKinney W., Xu J. (2018). Data Analysis Using Python.

[16] Du Y., Zhi J., Huang J., Xiang Z., He S., Wang J. (2020). Research on the innovative design of longitudinal sleeper compartments based on passenger satisfaction. Mech. Des. Res..

[17] Zhang L., Du Y., Chen F., Ma W. (2019). Analysis of hidden dangers in the use of outdoor fitness equipment based on FMEA and FTA. Mach. Manag. Dev..

[18] Weiye Health Technology (Longnan) Co., Ltd. (2016). A Fitness Equipment Damper.

